# The Progress of Lung Surgery

**Published:** 1913-02-15

**Authors:** 


### NEW DEVELOPMENTS IN MEDICINE.

### I. The Progress of Lung Surgery.

Fifteen years ago the surgery of the lungs was confined to timid ventures upon abscesses. Beyond the development of the surgery for empyema, in which the name of Reich, Estlander, and Esmarch are associated with that honour which belongs to the pioneer, little had been done in a radical manner. Indeed, to many surgeons the lung was taboo. Experiments upon it were for the most part failures, although some workers had already demonstrated that with proper aseptic precautions the thoracic organs could be attacked with success. Then the surgical world was startled by the report of a trial of a young German surgeon who had had the courage to operate on a patient, a girl, with advanced tuberculosis of the right apex. He had successfully removed the affected piece of lung tissue, but the patient died several days afterwards from shock. Her relatives brought an action against the surgeon, who was placed on his trial for malpraxis. In the course of the trial expert evidence was given to the effect that such an operation was hazardous in the extreme; that it was, in fact, in the then stage of surgical knowledge an unjustifiable operation. In the end the operator was found guilty and sentenced to imprisonment. The action lost him his practice, but it drew the attention of surgeons the world over to the possibilities of lung surgery. From that stage the progress was rapid, and crowned with the most remarkable results. A German operator, Gluck, excised a huge pulmonary sarcoma; a Swedish surgeon removed a foreign body deeply impacted in the right bronchus; a French operator evacuated a large intralobar abscess; an Italian successfully repeated the operation of excising a phthisical cavity at the apex of one lung. Sauerbruch, stimulated thereto by his master Mickulicz, investigated the influence of air pressure and devised his well-known high-pressure cabinet, which makes operations on the lung tissue possible without the risk of incurring that collapse which was the terror of operators. Braun, a few months later, constructed his improved low-pressure apparatus. Both these two types of special operating rooms are now used in various institutions. The German hospital at New York has a fine installation for pulmonary work specially designed by the late Dr. Beck, himself a pioneer in this special branch of surgery. University College Hospital in London has a less complete high-pressure cabinet in which several excellent operations have already been done. On the Continent nearly every important hospital possesses these special operating chambers for lung work, the fitting and building of which necessitates a comparatively large outlay of money.

In 1903 appeared Garre and Quincke's epoch-making "Grundriss der Lungenchirurgie," a book that dealt exclusively with the development of pulmonary surgery, and that gave the first clear indications for operative interference, and collected the various cited cases for reference. Since then the surgery of the lungs has progressed immensely. Trendelenburg showed how it is possible to operate on the pulmonary artery and take from it a thrombus, an operation which has now been done some eighteen times with varying degrees of success. Garre himself proved how it is possible, with the help of these special pressure cabinets, to undertake the most extensive resections of lung tissue. Not only a tuberculous cavity but entire lobes and portions of the lobes of the lungs have been removed; in one case almost the whole of one lung has been excised and the patient recovered. A new operation, dorsal bronchotomy, has been done to obtain an air-way in cases where the trachea was blocked and tracheotomy could not be done. The thoracic part of the œsophagus has been resected, and tumours from the mediastinum have been removed by thoracic surgery. Great helps in diagnosis and treatment have been the bronchoscope and tracheoscope, which render it possible for the operator to see clearly into the air-ways and to remove or localise obstructions. How immense has been the progress all along the line is evident from a perusal of Garre and Quincke's latest work, "Lungenchirurgie," published by Gustav Fischer, Jena, price 8s. 6d. net. This surgeon and physician, working in collaboration, have specialised in lung surgery, and the results of their collaboration are remarkable in view of the great difficulties which they have had to overcome. The initial chapters of their book deal with the various methods of operation.

It is this part especially that is of interest to the hospital worker. We cannot blink the fact that the progressive advance of modern medicine and the discovery of new methods and new procedures, which demand for their successful execution new and expensive appliances and a specially trained staff, have a high institutional importance. Hospital workers must realise that if their institutions are to be up to date and efficient it is necessary that they should fall into line with progressive hospitals and enable their surgeons and physicians to utilise these new discoveries in the best possible manner for the advantage of the patient. The installation of elaborate pressure cabinets is not always necessary—this latest book shows how much can be done without these aids—but no modern hospital can claim to be fully equipped unless it possesses a complete armamentarium for the lung surgeon. There are special and expensive instruments needed for dealing with the friable and delicate tissues of the lung; the nursing staff needs special training in the after-treatment of operated cases; the diagnostic instrumentarium entails considerable expenditure. But all these extra expenses must be cheerfully faced, for lung surgery is now an acknowledged branch of the art, and a patient who comes into hospital with an operable lung tumour has the right to demand that the hospital shall attempt to do for him what surgery elsewhere has successfully done for other patients similarly afflicted. The question still remains: In how far are intra-thoracic lesions operable? There are some old-fashioned surgeons who refuse to admit that all this advance is progress, and who sneer at what they term mischievous interference. But no one can overlook the success that has been attained in certain definite classes of cases. Thus, in the case of pulmonary cysts, especially those due to echinococcus, in actinomycosis of the lung, in circumscribed tubercular lesions, in pulmonary abscesses, in trauma of the lungs, in hernia, in the case of foreign bodies deeply impacted in the airway or penetrating into the lung tissue itself, in tumours, whether malignant or innocent, and possibly also in cases of advanced bronchiectasis and emphysema, lung surgery may legitimately be considered. But lung surgery itself is only a branch of the larger section of intra-thoracic surgery—that is, treatment by surgical means of lesions of the organs contained in the chest cavity. Therefore the surgeon will have to consider the advisability of dealing with affections of the thoracic œsophagus, of the mediastina, of the thoracic duct, of the large vessels, of the heart and the heart sac, and of the investing membranes. Formerly this field was outside the limits of legitimate surgery; to-day it is well within it, and although much has still to be done before we can lay claim to knowing exactly what lesions are fit for treatment and what lesions are not, we have already sufficient experience of detail to attempt to cure diseases of these organs which two decades ago we would have regarded as hopeless. The crux of the matter is that intra-thoracic surgery is no longer speculative and experimental; it has become definite, and must be recognised as such by the hospital worker just as he has come to recognise $x$-ray work, radium treatment, and massage.

